# Exercise-induced myokine irisin protects against obesity-associated ischemic stroke via integrin αVβ5 signaling

**DOI:** 10.3389/fphar.2026.1893339

**Published:** 2026-08-05

**Authors:** Eun Bi Ma, Seung Jun Hong, Hyangju Kang, Eun-Ju Sohn, Kang-Ho Choi, Joo Young Huh

**Affiliations:** 1 College of Pharmacy, Chung-Ang University, Seoul, Republic of Korea; 2 Department of Neurology, Chonnam National University Hospital, Chonnam National University Medical School, Gwangju, Republic of Korea; 3 Department of Global Innovative Drugs, The Graduate School of Chung-Ang University, Seoul, Republic of Korea; 4 BioApplications Inc., Pohang, Republic of Korea

**Keywords:** irisin, ischemic stroke, myokine, neuroinflammation, neuroprotection, obesity, integrin αVβ5

## Abstract

Ischemic stroke in the context of obesity represents a growing clinical challenge, yet obesity-specific neuroprotective strategies remain largely unexplored. Here, we investigated whether exercise-induced irisin, a myokine generated by proteolytic cleavage of fibronectin type III domain-containing protein 5 (FNDC5) in skeletal muscle, confers neuroprotection in a high-fat diet (HFD)-induced obese mouse model of transient middle cerebral artery occlusion (MCAO). Treadmill exercise reduced infarct size and restored circulating and cerebral FNDC5/irisin levels that were suppressed by obesity and ischemic injury. Intraperitoneal administration of recombinant irisin recapitulated these protective effects, reducing infarct volume and neurological deficits while upregulating the tight junction proteins occludin and claudin-5, suppressing microglial activation, promoting M2 macrophage polarization, and inhibiting NLRP3 inflammasome-mediated pyroptosis, as evidenced by reductions in cleaved caspase-1 and gasdermin D protein. Irisin treatment also significantly increased the protein expression of its receptor, integrin αVβ5. Conversely, genetic deletion of FNDC5 exacerbated ischemic outcomes, disrupted blood–brain barrier integrity, aggravated neuroinflammation, and reduced integrin αVβ5 expression, collectively mirroring the pathological features reversed by exogenous irisin. Pharmacological blockade of integrin αVβ5 with cilengitide abolished the neuroprotective effects of irisin, establishing the receptor-dependent nature of its actions. These findings demonstrate that the FNDC5/irisin/integrin αVβ5 axis functions as a receptor-dependent, exercise-inducible neuroprotective pathway in obesity-associated ischemic stroke, and identify recombinant irisin as a promising ““exercise mimetic”“ with therapeutic potential for this high-risk population.

## 1 Introduction

Stroke remains one of the leading causes of death and long-term disability worldwide, imposing a staggering burden on individuals, healthcare systems, and economies (G.B.D.S. Collaborators, 2021). Despite decades of intensive research, the therapeutic options for ischemic stroke remain critically limited. Intravenous thrombolysis and mechanical thrombectomy represent the only proven reperfusion strategies, yet their narrow therapeutic time windows and stringent eligibility criteria leave the vast majority of patients without effective treatment (Magoufis et al., 2021; Wechsler et al., 2021). Furthermore, acute reperfusion alone often fails to halt the secondary injury cascade, including oxidative stress, blood–brain barrier (BBB) disruption, and robust neuroinflammation, within the surrounding penumbral tissue. Consequently, many stroke survivors suffer from persistent motor and cognitive impairments (Al-Qazzaz et al., 2014; Langhorne et al., 2009), highlighting an urgent need for novel neuroprotective strategies.

This challenge is further compounded by the rapidly expanding global obesity epidemic, which now affects billions of individuals worldwide and is projected to rise continuously (Collaboration, 2024). This widespread metabolic burden not only increases ischemic stroke incidence but also independently exacerbates brain injury (Chen et al., 2018). Obesity is characterized by chronic, systemic inflammation driven by changes in adipokine secretion and increased circulating pro-inflammatory cytokines, emerging as a critical independent modifier of stroke pathology (Bushnell et al., 2024; Le Thuc and Garcia-Caceres, 2024). This systemic metabolic stress adversely affects the central nervous system even before ischemic events occur, impairing the integrity of the BBB and chronically activating resident microglia and the NLRP3 inflammasome (Vand et al., 2011; Zhao et al., 2024), thereby driving the brain into a pro-inflammatory, pyroptosis-prone state (Le Thuc and Garcia-Caceres, 2024; Ramirez-Carreto et al., 2023). Consequently, when cerebral ischemia occurs in an obese state, this pre-existing neurovascular fragility triggers an excessive neuroinflammatory response, leading to accelerated cell death, increased infarct volume, and poorer functional recovery (Bukhari et al., 2023; Kernan et al., 2013; Yousufuddin and Young, 2019). Despite the high risk of disability after stroke in the obese population, obesity-specific neuroprotective strategies have not yet been sufficiently studied. This translational gap further underscores the imperative need for novel, targeted approaches capable of mitigating secondary brain damage after acute reperfusion and improving long-term neurological recovery in this metabolically vulnerable population.

Physical exercise is a well-documented intervention for both stroke prevention and post-stroke rehabilitation, reducing stroke risk through improvements in blood pressure, lipid profiles, insulin sensitivity, and systemic inflammation, while promoting neuroplasticity and functional recovery following stroke (Gallanagh et al., 2011; Prior and Suskin, 2018). These multifaceted benefits are mediated not only by hemodynamic adaptations but also by the secretion of bioactive peptides from contracting skeletal muscle, a class of signaling molecules termed myokines (Bay and Pedersen, 2020). Crucially, several myokines can cross the blood–brain barrier (BBB) and exert direct effects on the central nervous system, thereby translating peripheral exercise signals into cerebral neuroprotection (Kostka et al., 2024).

Among candidate myokines, irisin has emerged as a particularly promising mediator, as it is uniquely positioned at the intersection of metabolic and neurological regulation. Irisin is produced by proteolytic cleavage of the membrane protein fibronectin type III domain-containing protein 5 (FNDC5) in response to exercise (Bostrom et al., 2012). Initially characterized for its role in driving the browning of white adipose tissue and enhancing systemic energy expenditure (Bostrom et al., 2012), irisin has since been recognized as a pleiotropic molecule with profound activity in the central nervous system, where it stimulates the expression of brain-derived neurotrophic factor (BDNF), promotes neurogenesis, and protects hippocampal neurons from apoptotic and excitotoxic injury (Liu et al., 2020). In the context of ischemic stroke, clinical studies have demonstrated that lower serum irisin at admission independently predicts poor functional outcomes and increased mortality in patients with acute ischemic stroke (Wu et al., 2019), while post-stroke depression is associated with lower circulating irisin levels (Tu et al., 2018). In experimental models of cerebral ischemia, exogenous irisin has been reported to reduce infarct injury and improve neurological outcomes, in association with activation of pro-survival signaling and neurotrophic responses (Asadi et al., 2018; Li et al., 2017). Of particular relevance, obesity and physical inactivity are associated with chronically reduced circulating irisin levels, suggesting that obese individuals may be especially vulnerable to ischemic injury in the setting of irisin deficiency (Hou et al., 2015; Moreno-Navarrete et al., 2013).

Recently, integrin αVβ5 has been identified as functional receptor for irisin, potentially mediating its downstream cellular effects (Kim et al., 2018). In a model of intracerebral hemorrhage, irisin was shown to inhibit microglia/macrophage pro-inflammatory polarization and promote the anti-inflammatory M2 phenotype through integrin αVβ5/AMPK signaling, and this neuroprotective effect was abolished by pharmacological blockade of the receptor with cilengitide (Wang et al., 2022a). However, despite these advances, the causal contribution of irisin signaling to ischemic stroke outcomes, particularly in the clinically relevant context of obesity, remains insufficiently defined. Furthermore, whether integrin αVβ5-dependent mechanisms mediate irisin’s protective effects specifically in ischemic brain injury has not been established.

To address these gaps, the present study employed a high-fat diet (HFD)-induced obese mouse model of transient middle cerebral artery occlusion (MCAO), utilizing a multi-pronged experimental approach. We investigated whether treadmill exercise mitigates obesity-exacerbated ischemic brain injury through enhanced FNDC5/irisin production, and whether recombinant irisin recapitulates these neuroprotective effects by examining infarct size, BBB integrity, microglial activation, macrophage polarization, and NLRP3 inflammasome-mediated pyroptosis. To further validate the role of endogenous irisin, FNDC5-deficient mice were employed as a genetic loss-of-function model. Finally, the receptor-dependence of irisin’s actions was established through pharmacological inhibition of integrin αVβ5 with cilengitide. Collectively, this study was designed to elucidate the FNDC5/irisin/integrin αVβ5 axis as a receptor-dependent neuroprotective pathway and to evaluate the therapeutic potential of irisin for obesity-associated ischemic stroke.

## 2 Materials and methods

### 2.1 Animal experiments

C57BL/6 male mice were purchased from Samtako Bio Korea (Osan, Korea). All animal experiments were performed in compliance with the care and use guidelines of Chonnam National University and were approved by the Institutional Animal Care and Use Committee (IACUC). Mice were housed in a pathogen-free animal facility maintained at 22 °C ± 2 °C and 55% ± 5% humidity under a 12-h light/dark cycle, with unrestricted access to food and water. Mice were fed either a normal chow diet (NCD; Rod Feed, DBL Co., Ltd., Eumseong, Republic of Korea) or a high-fat diet (HFD; D12492, Research Diets, New Brunswick, NJ, United States). By energy, the NCD provided approximately 24% protein, 10% fat, and 66% carbohydrate, whereas the HFD provided 20% protein, 60% fat, and 20% carbohydrate. Mice were maintained on the assigned diets for 8 weeks before MCAO. This window was chosen on two grounds: an 8-week high-fat diet reliably induces obesity and insulin resistance in C57BL/6 mice (de et al., 2009), and it yields a body weight suited to the intraluminal filament model, in which filament diameter must match the body-weight–dependent caliber of the carotid/cerebral arteries for reproducible occlusion (Llovera et al., 2021; Morris et al., 2016). Obesity was defined as a significant body-weight increase versus age-matched NCD controls. Prior to sacrifice, mice were anesthetized with 3% isoflurane (Hana Pharm Co., Ltd., Seoul, Korea) in an induction chamber until loss of the pedal withdrawal reflex and subsequently euthanized by cervical dislocation.

For the exercise study, mice were randomly assigned to three groups: NCD, HFD, and HFD + E. After 4 weeks of HFD feeding, mice in the HFD + E group underwent 4 weeks of treadmill exercise (Jeungdo B&P Co., Ltd., Seoul, Korea) while continuing on the HFD. Exercise was performed 5 days per week, beginning at 10–12 m/min for 10 min and progressing to 20 m/min for 20 min per session. Following completion of the exercise protocol, all three groups underwent transient middle cerebral artery occlusion (MCAO) surgery.

For the irisin treatment study, mice were fed an HFD for 8 weeks prior to MCAO. Recombinant irisin produced in *Nicotiana benthamiana* (Ma et al., 2024) was reconstituted in PBS and tested at three doses (250, 750, and 1000 μg/kg). Mice received intraperitoneal injections of irisin or PBS immediately before MCAO.

For the FNDC5 KO study, age-matched wild-type (WT) and FNDC5 knockout (KO) mice (Cyagen, Santa Clara, CA, United States) (Liu et al., 2023) were fed an HFD for 8 weeks and subjected to MCAO. For the rescue experiment, HFD-fed WT and FNDC5 KO mice received an intraperitoneal injection of recombinant irisin (750 μg/kg) or PBS immediately before MCAO.

For integrin αVβ5 inhibition, cilengitide (10 mg/kg, Selleck, Houston, TX, United States) dissolved in DMSO was administered via intraperitoneal injection 2 h prior to MCAO.

### 2.2 Transient middle cerebral artery occlusion

To model cerebral ischemia/reperfusion injury, mice were subjected to transient middle cerebral artery occlusion (MCAO) surgery. In all experiments, a sham group consisting of NCD-fed mice that underwent the same surgical procedure without filament insertion was included as a baseline control. Anesthesia was induced with 3% isoflurane in a 30:70 oxygen/nitrous oxide mixture and maintained with 2% isoflurane after the animals were placed in a stereotaxic frame. Body temperature was maintained at 37 °C throughout the procedure using a heating pad. An incision was made along the midline of the neck, followed by dissection to reveal the common carotid artery (CCA), internal carotid artery (ICA), and external carotid artery (ECA). The CCA and ECA were exposed and tied off using a loop. Subsequently, a 6-0 monofilament nylon suture (Doccol Co., Redlands, CA, United States) was inserted into the CCA, advanced into the ICA to occlude the middle cerebral artery, and withdrawn after 60 min to allow reperfusion.

### 2.3 Neurological score assessment and cerebral infarct volume measurement

Neurological deficits in mice were monitored daily for 3 days following middle cerebral artery occlusion. Neurological deficits were assessed using seven specific criteria. The evaluation criteria include body symmetry, gait, climbing, circling behavior, front limb symmetry, compulsory circling, and whisker response. The scoring scale ranged from 0 (no detectable deficits) to 28 (absence of spontaneous motor activity and loss of consciousness), and all scores were recorded (Llovera et al., 2021). Three days after undergoing MCAO surgery, the mice were euthanized, and their brains were harvested, and cut into 2-mm coronal sections. The brain slices were incubated with 2% 2,3,5-triphenyltetrazolium chloride (TTC; Sigma-Aldrich, St. Louis, MO, United States) at 37 °C for 10 min. Stained sections were photographed, and the infarct area was quantified using ImageJ software (NIH, Bethesda, MD, United States).

### 2.4 RNA isolation and real-time PCR

Total RNA was extracted from mouse brain tissues using TRIzol (MRC, Cincinnati, OH, United States) according to the manufacturer’s instructions. The reverse transcription of total RNA into cDNA was carried out using TOPscript™ RT DryMIX (Enzynomics, South Korea). The TOPreal SYBR Green PCR Kit was used to conduct real-time polymerase chain reaction (Enzynomics) on the Rotor-Gene Q PCR cycler (QIAGEN, Hilden, Germany). Real-time PCR was performed under the following conditions: initial denaturation at 95 °C for 15 min, then 40 to 45 cycles of 95 °C for 10 s, 62 °C for 15 s, and 72 °C for 20 s 18S ribosomal RNA was used as the internal control for normalization of mRNA levels.

### 2.5 Western blot

Western blot analysis was carried out according to the previously described method (Ma et al., 2019). Briefly, total protein from mouse brain samples was extracted using RIPA buffer (Thermo Scientific, Rockford, IL, United States) and the protein concentration was measured with the Pierce™ BCA Protein Assay Kit (Thermo Scientific). Proteins were separated by SDS-PAGE, transferred to a PVDF membrane (Cytiva, Uppsala, Sweden), and blocked with 5% skim milk. After incubating with primary and secondary antibodies, protein bands were detected using Amersham™ ECL Select™ Western Blotting Detection Reagent (Cytiva). Protein expression levels were quantified with the Fujifilm LAS 4000 Gel Luminescent Image Analyzer (Fujifilm, Tokyo, Japan).

### 2.6 Enzyme-linked immunosorbent assay

Blood samples for plasma analysis were obtained through cardiac puncture immediately after sacrifice. Plasma was then isolated by centrifugation at 3,000 rpm for 20 min at 4 °C and preserved at −80 °C until further analysis. Plasma irisin levels were measured using a commercially available ELISA kit (R&D Systems, Minneapolis, MN, United States), according to the manufacturer’s instructions. Briefly, plasma samples and standards were added to 96-well ELISA plates pre-coated with the capture antibody. After a 2-h incubation at room temperature, the plates were washed, and the detection antibody was added and incubated for another 2 h at room temperature. After incubation, streptavidin-horseradish peroxidase (SA-HRP) solution was added, followed by tetramethylbenzidine (TMB) substrate solution. Plasma concentrations were determined by measuring absorbance at 450 nm using a microplate reader (Synergy HTX; BioTek, Winooski, VT, United States).

### 2.7 Immunofluorescence assay

Brain tissues were formalin-fixed and paraffin-embedded prior to immunofluorescence staining. Paraffin-embedded tissue sections were deparaffinized in xylene (DUKSAN Co., Ltd., Ansan, Korea) and rehydrated through graded series of ethanol solutions. Antigen retrieval was carried out in 10 mM citrate buffer containing 2 mM EDTA and 0.05% Tween-20 (pH 6.0) using a pressure cooker for 15 min. To minimize non-specific binding, slides were incubated in 3% bovine serum albumin for 15 min in a humid chamber. Tissue sections were then incubated with a primary antibody against IBA1 (Fujifilm Wako Pure Chemical Corporation, Osaka, Japan) overnight at 4 °C in a humid chamber. The following day, the tissue sections were washed twice with 1× TBST for 10 min each. Sections were subsequently incubated with Alexa Fluor 488–conjugated anti-rabbit IgG (1:400, Thermo Scientific) for 1 h at room temperature in the dark. Nuclear counterstaining was carried out using DAPI (Invitrogen, Waltham, MA, United States) for 10 min. Slides were then examined by fluorescence microscopy (Zeiss LSM-510; Jena, Germany).

### 2.8 Statistical analysis

Statistical analyses were performed with StatView v5.0 software (SAS Institute, Inc. Cary, NC, United States), and data visualization was performed using GraphPad Prism v8.0 software (San Diego, CA, United States). The data are represented as the mean ± standard error of the mean (SEM). Data were analyzed using one-way analysis of variance (ANOVA) or a two-tailed unpaired t-test where appropriate. Statistical significance was defined as a *P* value <0.05.

## 3 Results

### 3.1 Exercise reduces ischemic brain injury in obese mice and enhances irisin production and FNDC5/irisin expression

**FIGURE 1:**
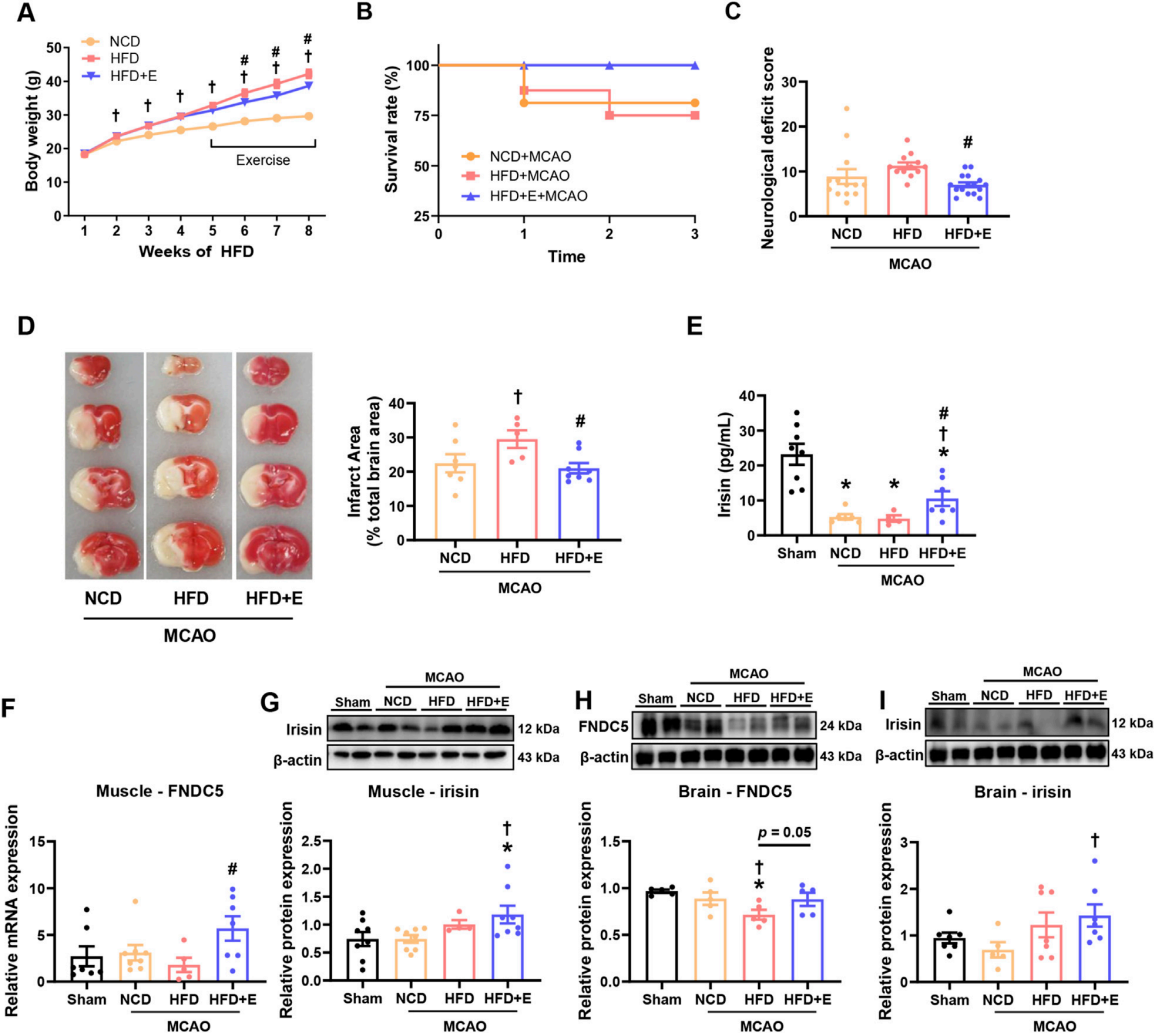
Exercise reduces infarct size and enhances irisin expression in a mouse model of ischemic stroke. C57BL/6 mice were fed either a normal chow diet (NCD) or high-fat diet (HFD) for 8 weeks and a group of mice underwent 4 weeks of treadmill exercise after 4 weeks of HFD feeding. After 8 weeks of HFD, mice were subjected to transient middle cerebral artery occlusion (MCAO) surgery. **(A)** Body weight measurements were collected over an 8-week of HFD period. **(B)** Post-MCAO surgery survival rate. **(C)** Neurological deficit evaluation following MCAO surgery. **(D)** TTC Staining to evaluate cerebral infarction size in brain tissue after MCAO surgery. **(E)** The secretion of irisin in blood was quantified by ELISA. **(F)** The mRNA expression of FNDC5 in muscle was quantified using real-time PCR. **(G)** The protein expression of irisin in muscle was quantified using western blot. **(H,I)** The protein expression of FNDC5 and irisin in the brain was quantified using western blot. *p < 0.05 vs. Sham (NCD-fed mice subjected to sham surgery), ^†^ *p* < 0.05 vs. NCD + MCAO, ^#^ *p* < 0.05 vs. HFD + MCAO.

To investigate the effect of exercise on ischemic stroke under metabolic stress, we employed an HFD–fed mouse model subjected to MCAO. As expected, mice in the HFD group exhibited significantly higher body weight compared with NCD mice (Figure 1A). The treadmill exercise group (HFD + E) showed markedly reduced body weight relative to the sedentary HFD group. At the end of the exercise training period, transient MCAO was performed to induce cerebral ischemia. Exercise restored the reduced survival rate within 3 days after MCAO surgery and showed a tendency to attenuate the neurological deficits elevated by HFD (Figures 1B,C). Consistently, TTC staining demonstrated that HFD feeding exacerbated ischemic brain injury, as evidenced by increased infarct size compared with NCD mice following MCAO, whereas exercise significantly attenuated infarct size in HFD-fed mice (Figure 1D).

To determine whether the exercise-induced neuroprotection was associated with the myokine irisin, we measured circulating irisin levels. Plasma irisin concentrations were significantly reduced following MCAO in both NCD and HFD groups compared with the NCD-fed sham group, but were restored by exercise (Figure 1E). In parallel, both FNDC5 mRNA and irisin protein expression were significantly upregulated in skeletal muscle in the exercise group (Figures 1F,G), suggesting that increased muscle FNDC5 expression contributes, at least in part, to elevated circulating irisin levels. Although irisin is predominantly expressed in skeletal muscle, emerging evidence suggests that it is also produced locally in the brain (Muzaffar et al., 2025). Consistently, brain FNDC5 protein expression was significantly reduced in HFD group and showed a tendency to increase in the exercise group (Figure 1H). Similar to muscle expression, brain irisin level was significantly upregulated in the exercise group compared with NCD control (Figure 1I). Together, these results demonstrate that while obesity exacerbates ischemic brain damage and reduces circulating irisin levels, exercise mitigates these effects potentially in association with enhanced FNDC5/irisin levels.

### 3.2 Irisin attenuates ischemic brain damage and induces FNDC5 and integrin αVβ5 expression in obesity-associated ischemic stroke

**FIGURE 2:**
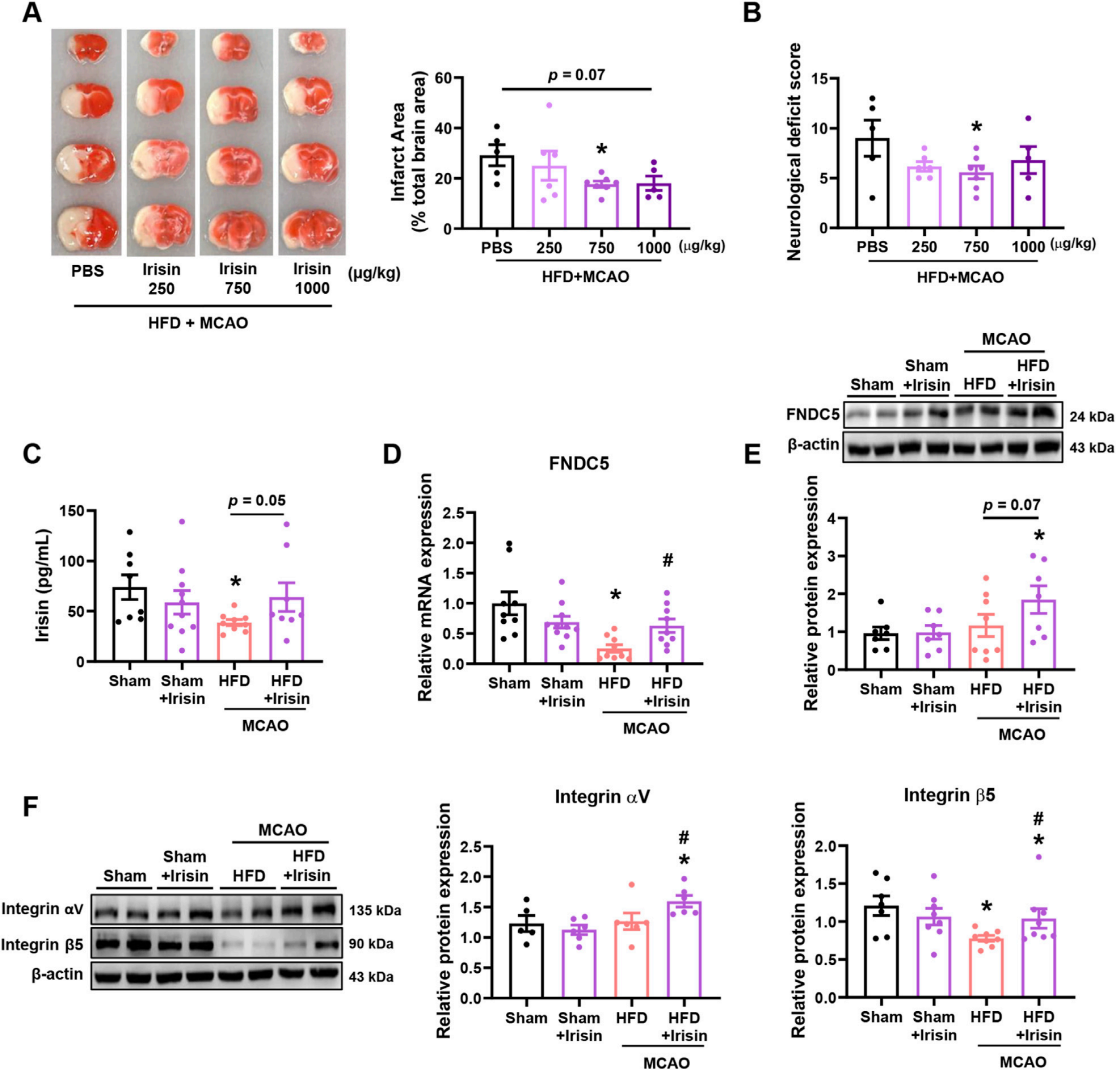
Assessment of recombinant irisin administration in an ischemic stroke mouse model. C57BL/6 mice were fed a high-fat diet (HFD) for 8 weeks and recombinant irisin was injected immediately before MCAO surgery. The effects of irisin on ischemic injury were evaluated using three different doses: 250, 750, 1000 μg/kg. **(A)** Quantification of cerebral infarction size and brain tissue staining following irisin treatment at multiple concentrations in an ischemic stroke model. **(B)** Neurological deficit assessment following MCAO surgery. **(C)** Irisin levels in the blood were measured using an ELISA assay. **(D,E)** The expression of FNDC5 mRNA and protein in the brain was analyzed using real-time PCR and western blot. **(F)** The expression of integrin αV and β5 proteins in the brain was determined by western blot. *p < 0.05 vs. Sham (NCD-fed mice subjected to sham surgery), ^#^ *p* < 0.05 vs. HFD + MCAO.

Since circulating irisin levels were reduced in MCAO mice and irisin expression in muscle and brain were markedly increased by exercise, we next investigated whether exogenous irisin treatment could act as an exercise mimetic to alleviate MCAO-induced brain injury in HFD mice. Mice were fed an HFD for 8 weeks, and recombinant irisin was administered intraperitoneally immediately prior to vascular occlusion. To determine the optimal dose, a dose–response analysis was performed using three concentrations of irisin (250, 750, and 1000 μg/kg). Both TTC staining and neurological deficit scores demonstrated that irisin at 750 μg/kg produced the most pronounced protective effects (Figures 2A,B); therefore, this dose was used for all subsequent experiments. Plasma irisin concentration, which was reduced in HFD + MCAO mice compared to sham mice, showed a tendency to increase following irisin treatment (Figure 2C). Notably, irisin administration significantly reversed the reduction in brain FNDC5 mRNA expression observed in HFD + MCAO mice (Figure 2D). Brain FNDC5 protein expression was also marginally increased in the irisin-treated group compared with the HFD + MCAO group (Figure 2E), suggesting a potential feed-forward mechanism. In parallel, irisin treatment significantly increased the protein expression of the irisin receptors integrin αV and β5 (Figure 2F). Among the two, only integrin β5 was significantly decreased in HFD + MCAO group compared with sham. Taken together, these results indicate that exogenous irisin administration recapitulates the neuroprotective effects of exercise and upregulates integrin αVβ5 receptor expression in the ischemic brain of obese mice, supporting the role of the FNDC5/irisin/integrin αVβ5 axis as a mediator of exercise-induced neuroprotection.

### 3.3 Irisin preserves blood–brain barrier integrity and suppresses microglial activation in obesity-associated ischemic stroke

**FIGURE 3:**
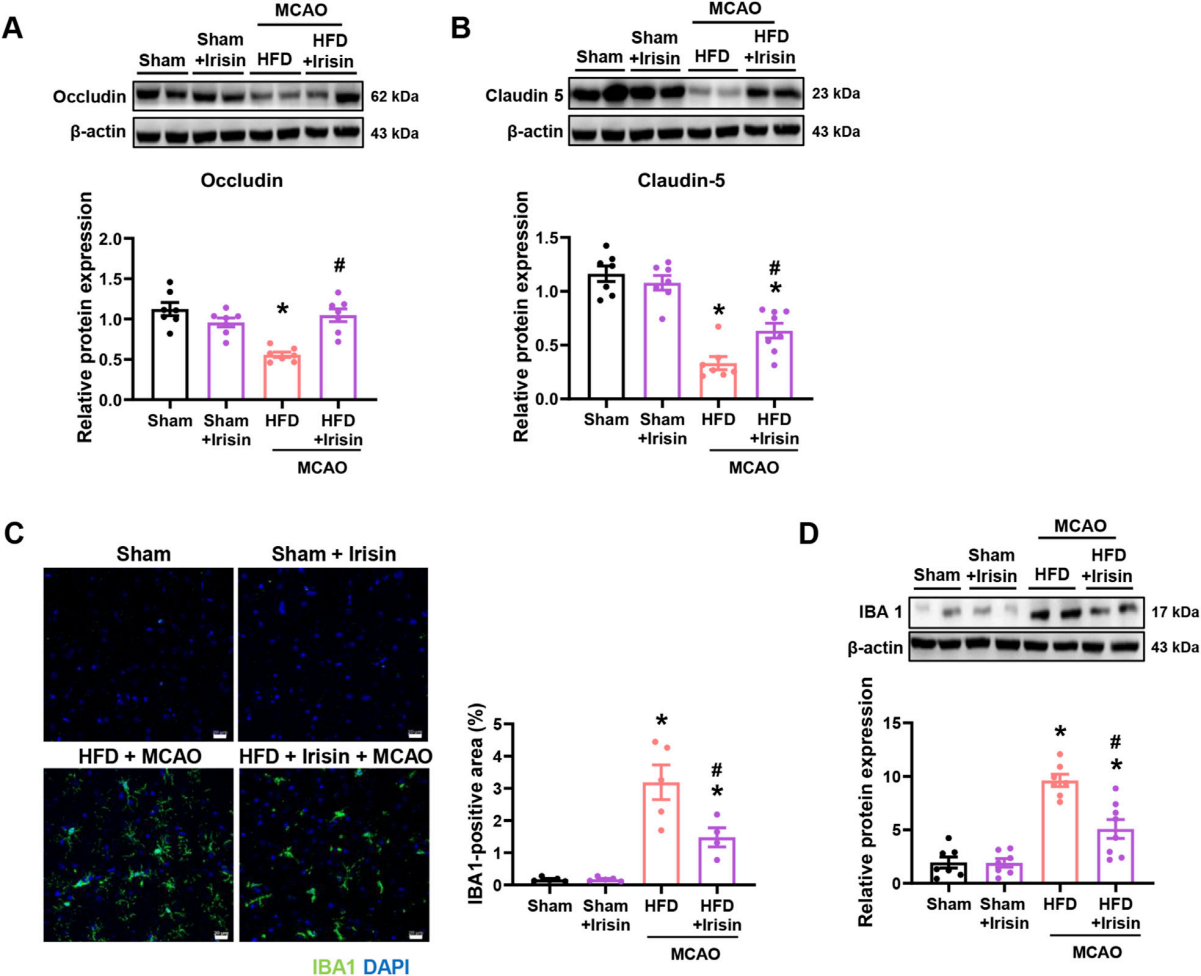
Recombinant irisin preserves blood–brain barrier integrity and suppresses microglial activation. Biomarkers of blood–brain barrier (occludin and claudin-5) and microglial activation (IBA1) were measured in obesity-associated MCAO mice treated with recombinant irisin (750 μg/kg, intraperitoneally, immediately before MCAO). Sham group fed NCD received the same surgical procedure without MCAO. **(A,B)** The protein expression of occludin and claudin-5 in the brain was assessed by western blot. **(C)** Immunofluorescence staining of IBA1 and subsequent quantification of the IBA1-positive area. Scale bar = 20 μm. **(D)** IBA1 expression in the brain was measured by western blot analysis. *p < 0.05 vs. Sham, ^#^ *p* < 0.05 vs. HFD + MCAO.

Because blood–brain barrier (BBB) disruption is a hallmark of ischemic stroke pathology and is critically amplified in the context of obesity-associated metabolic inflammation, we first examined whether irisin could preserve BBB integrity. The expression of the tight junction proteins occludin and claudin-5 was markedly reduced in the brains of HFD + MCAO mice compared with sham controls, indicating substantial BBB disruption under obese ischemic conditions. Irisin administration significantly reversed these reductions, restoring occludin and claudin-5 protein levels (Figures 3A,B), suggesting that irisin protects the structural integrity of the BBB following cerebral ischemia in obese mice.

We next examined the effect of irisin on microglial activation, a key cellular mediator of post-ischemic neuroinflammation, tissue damage, and regenerative processes (Wang et al., 2022b). Immunofluorescence staining revealed a significant increase in the IBA1-positive area in the ischemic hemisphere of HFD + MCAO mice compared with sham controls, which was markedly reduced by irisin treatment (Figure 3C). Consistent with this, western blot analysis confirmed that IBA1 protein expression was significantly elevated in HFD + MCAO mice and was significantly reduced following irisin administration (Figure 3D). Collectively, these results indicate that irisin preserves BBB structural integrity and suppresses microglial activation in the obese ischemic brain, suggesting a coordinated attenuation of both vascular and cellular components of post-ischemic neuroinflammation.

### 3.4 Irisin promotes anti-inflammatory macrophage polarization and inhibits NLRP3 inflammasome-driven pyroptosis

**FIGURE 4:**
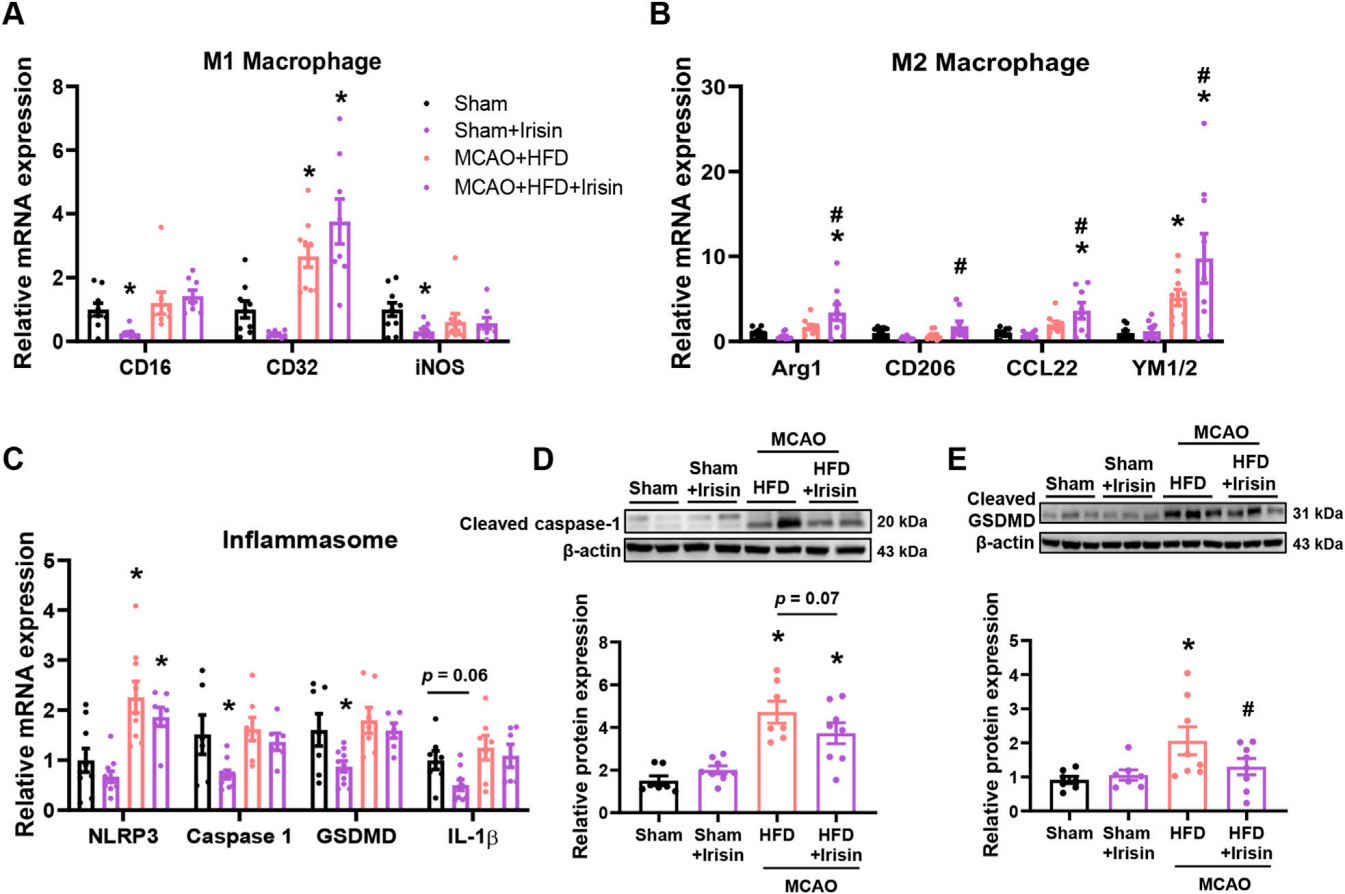
Irisin promotes M2 polarization and suppresses NLRP3 inflammasome activation. Biomarkers of M1 and M2 macrophage and NLRP3 inflammasome were measured in obesity-associated MCAO mice treated with recombinant irisin (750 μg/kg, intraperitoneally, immediately before MCAO). Sham group fed NCD received the same surgical procedure without MCAO. **(A,B)** The mRNA expression of M1 and M2 phenotype markers in the brain was evaluated using real-time PCR. **(C)** Inflammasome-related marker mRNA levels in the brain were evaluated using real-time PCR. **(D,E)** Protein expression of cleaved caspase-1 and cleaved GSDMD in the brain was evaluated using western blot. *p < 0.05 vs. Sham, ^#^ *p* < 0.05 vs. HFD + MCAO.

Macrophage polarization toward pro-inflammatory (M1) or anti-inflammatory (M2) phenotypes critically influences neuroinflammatory responses and tissue repair following ischemic stroke (Xiong et al., 2016), and therefore we evaluated whether irisin can modulate macrophage polarization. Gene expression analysis revealed that irisin did not significantly alter the expression of CD16, CD32, and iNOS M1 macrophage markers in HFD + MCAO mice, although a significant reduction was observed in irisin-treated sham mice compared with untreated sham controls (Figure 4A). In contrast, M2 macrophage markers Arg1, CD206, CCL22, YM1/2 were all significantly upregulated by irisin in HFD + MCAO mice (Figure 4B), demonstrating a selective shift toward an anti-inflammatory and reparative macrophage phenotype in the ischemic setting.

We further investigated the NLRP3 inflammasome pathway, which plays a central role in macrophage-mediated inflammatory amplification and pyroptotic cell death, and is closely linked to stroke pathology (Fusco et al., 2020; Tong et al., 2015). Gene expression showed that irisin reduced basal caspase-1, GSDMD, and IL-1β mRNA expression in sham group, but did not alter mRNA expression in the HFD + MCAO groups (Figure 4C). NLRP3 mRNA expression was significantly upregulated in both HFD + MCAO groups regardless of irisin treatment, with no significant difference observed between the two groups. At the protein level, cleaved caspase-1 was significantly increased in HFD + MCAO mice compared with sham controls and showed a decreasing trend following irisin treatment (Figure 4D). Similarly, the cleaved form of gasdermin D (GSDMD), the terminal pore-forming effector of NLRP3 inflammasome-mediated pyroptosis, was significantly elevated by HFD + MCAO and was significantly reduced by irisin administration (Figure 4E). The significant suppression of cleaved GSDMD protein despite the absence of transcriptional changes in MCAO groups suggests that irisin’s anti-pyroptotic effects operate primarily at the post-translational level in the context of acute ischemic injury. Collectively, these findings demonstrate that irisin attenuates ischemic stroke-induced neuroinflammation by promoting M2 macrophage polarization and inhibiting NLRP3 inflammasome-mediated pyroptosis.

### 3.5 FNDC5 deficiency exacerbates ischemic stroke

**FIGURE 5:**
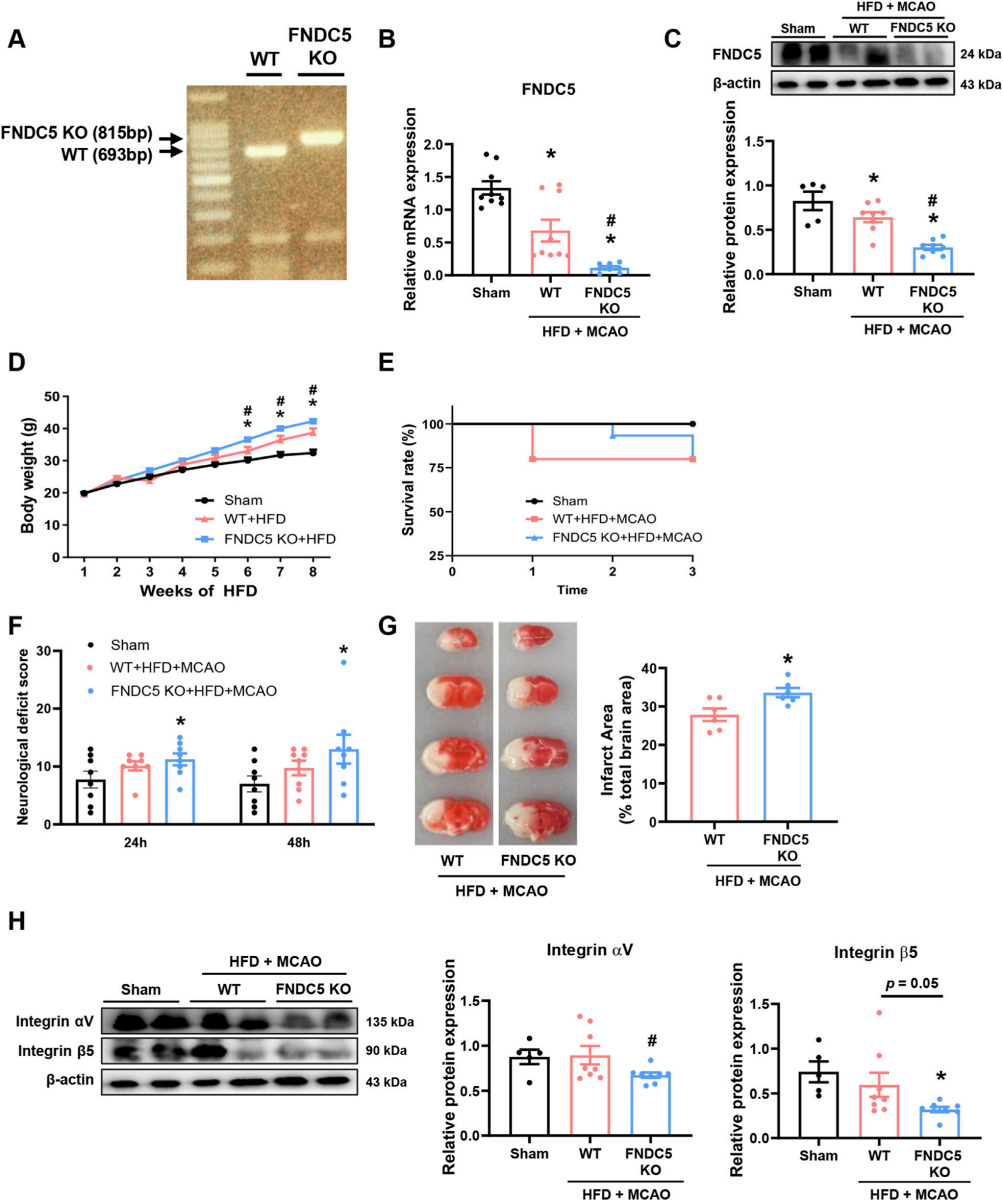
The absence of FNDC5 significantly aggravates the outcomes of ischemic stroke. Wild-type (WT) and FNDC5 knockout (KO) mice were fed high-fat diet (HFD) for 8 weeks and subjected to MCAO surgery. **(A)** FNDC5 deficiency was confirmed by PCR genotyping. **(B,C)** FNDC5 mRNA and protein expression in the brain were quantified using real-time PCR and western blot. **(D)** Body weight tracking was performed over the course of 8 weeks of HFD. **(E)** Survival outcomes following MCAO surgery. **(F)** Neurological deficit evaluation following MCAO surgery. **(G)** Measurement of cerebral infarction size and brain tissue staining with TTC following MCAO surgery. **(H)** The expression of integrin αV and β5 proteins in the mouse brain was determined by western blot analysis. *p < 0.05 vs. Sham (NCD-fed mice subjected to sham surgery), ^#^ *p* < 0.05 vs. WT + HFD + MCAO.

Having demonstrated that exogenous irisin confers neuroprotection in obesity-associated ischemic stroke, we next determined whether deficiency of endogenous irisin contributes to the progression of ischemic brain injury. Genotyping was first performed to confirm FNDC5 deletion in knockout mice (Figure 5A). In the brain, both FNDC5 mRNA and protein expression were markedly reduced in FNDC5-deficient mice compared with WT mice, confirming effective knockout (Figures 5B,C). WT and FNDC5-deficient mice were fed an HFD for 8 weeks, and prior to MCAO, FNDC5-deficient mice exhibited significantly higher body weight than WT mice, indicating that endogenous irisin deficiency further exacerbates HFD-induced obesity (Figure 5D). Although survival rates did not differ significantly between groups (Figure 5E), FNDC5-deficient mice displayed significantly increased neurological deficit scores at 24 and 48 h following MCAO compared with WT mice (Figure 5F). Consistent with these functional deficits, TTC staining revealed a significantly larger cerebral infarct volume in FNDC5-deficient mice (Figure 5G). Notably, FNDC5 deficiency resulted in a reduction in the protein expression of both integrin αV and integrin β5 in brain tissue compared with WT mice (Figure 5H), suggesting that endogenous irisin signaling is required for the maintenance of basal integrin αVβ5 receptor expression in the ischemic brain. These findings collectively establish that endogenous FNDC5/irisin plays a protective role in obesity-associated ischemic stroke, and that its deficiency recapitulates and amplifies the pathological features attenuated by exogenous irisin administration.

### 3.6 FNDC5 deficiency exacerbates MCAO-induced BBB disruption and inflammatory responses

**FIGURE 6:**
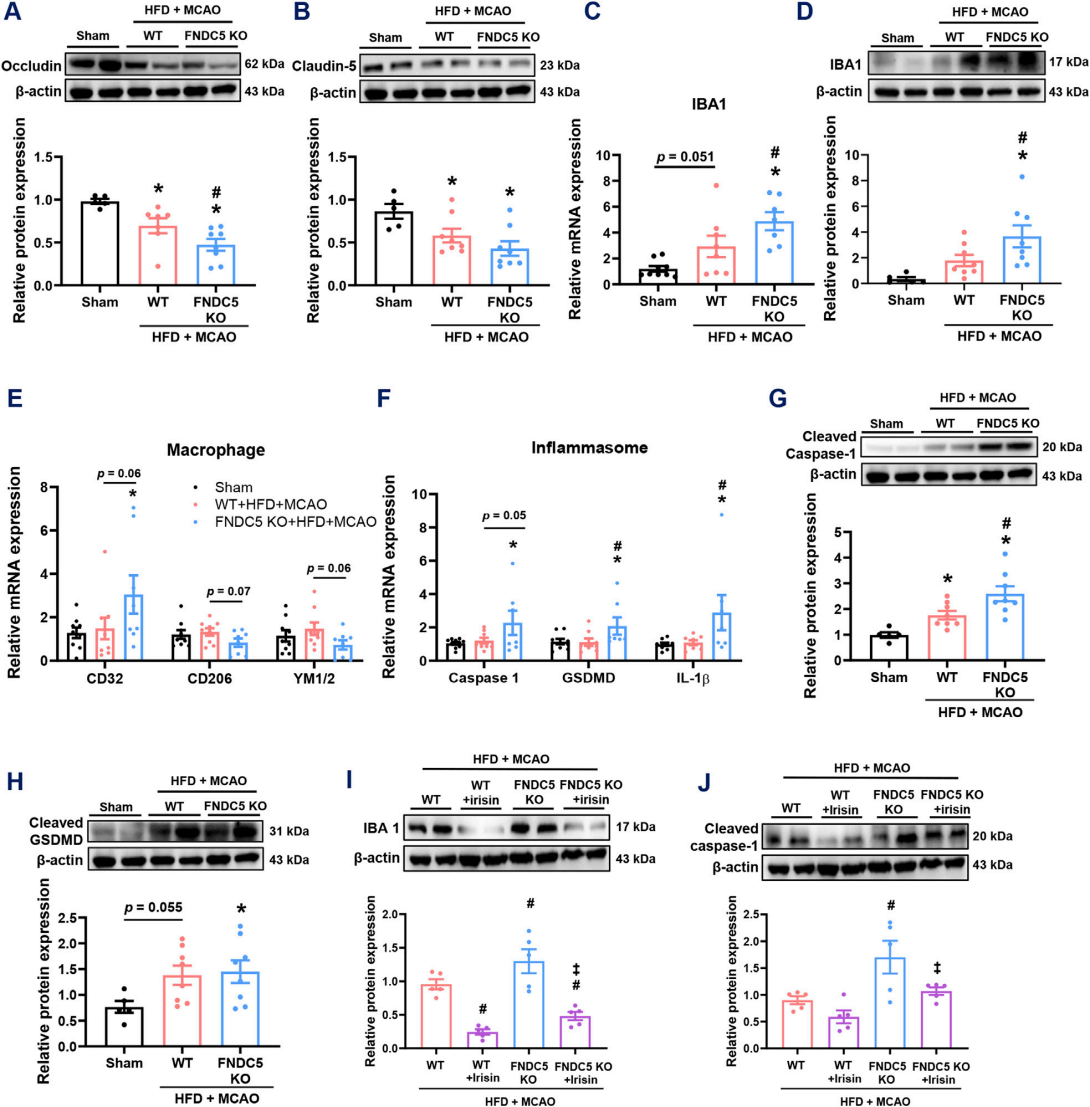
The lack of FNDC5 amplifies BBB injury and increases neuroinflammation. Wild-type (WT) and FNDC5 KO mice fed HFD were subjected to MCAO surgery, and biomarkers of BBB, microglial activation, macrophage activation, and inflammasome were measured. **(A,B)** The protein levels of occludin and claudin-5 in the brain were quantified by western blotting. **(C,D)** IBA1 expression in the brain was measured by real-time PCR and western blot analysis. **(E)** In the brain, the mRNA expression levels of macrophage markers were analyzed by real-time PCR. **(F)** The expression of inflammasome markers was assessed by real-time PCR. **(G,H)** The expression levels of cleaved caspase-1 and cleaved GSDMD in the brain were determined using western blot analysis. *p < 0.05 vs. Sham (NCD-fed mice subjected to sham surgery), ^#^ *p* < 0.05 vs. WT + HFD + MCAO. **(I,J)** WT and FNDC5 KO mice fed HFD received intraperitoneal injection of recombinant irisin (750 μg/kg) or vehicle immediately prior to MCAO surgery. The expression levels of IBA1 and cleaved caspase-1 were determined using western blot analysis. ^#^ *p* < 0.05 vs. WT + HFD + MCAO, ^‡^ *p* < 0.05 vs. FNDC5 KO + HFD + MCAO.

We next examined whether FNDC5 deficiency affects BBB integrity and inflammatory responses in an obesity-associated ischemic stroke. Protein expression of occludin and claudin-5 was reduced by HFD + MCAO and was further decreased by FNDC5 deficiency (Figures 6A,B). In contrast, IBA1 mRNA and protein expression were consistently increased in FNDC5 KO mice compared with WT mice (Figures 6C,D), indicating that FNDC5 deficiency aggravates microglial activation in the ischemic brain. Among macrophage polarization markers, the M1 macrophage marker CD32 showed a tendency toward increased expression, whereas the M2 macrophage markers CD206 and YM1/2 were modestly but consistently reduced by FNDC5 deficiency compared with control mice (Figure 6E), indicating a shift toward a pro-inflammatory macrophage phenotype. We further investigated whether FNDC5 deficiency influences inflammasome activation. The mRNA expression of inflammasome-related genes, including caspase-1, IL-1β, and GSDMD, was increased in FNDC5-deficient mice compared with WT mice (Figure 6F). Consistently, at the protein level, cleaved caspase-1 expression was significantly upregulated in FNDC5-deficient mice, while cleaved GSDMD protein expression showed an increasing trend that did not reach statistical significance (Figures 6G,H).

To further verify that the aggravated phenotype of FNDC5 KO mice is attributable to irisin deficiency, recombinant irisin was administered to HFD-fed FNDC5 KO mice immediately prior to MCAO. Exogenous irisin significantly reduced IBA1 and cleaved caspase-1 protein expression in FNDC5 KO mice, indicating effective suppression of microglial activation and inflammasome activation even in the absence of endogenous FNDC5 (Figures 6I,J). Collectively, these findings demonstrate that loss of endogenous FNDC5 aggravates MCAO-induced BBB disruption, skews macrophage polarization toward a pro-inflammatory state, and promotes inflammasome activation in obesity-associated ischemic stroke, and that these pathological features are partially reversible by exogenous irisin administration.

### 3.7 Irisin receptor inhibition leads to deterioration of stroke outcomes

**FIGURE 7:**
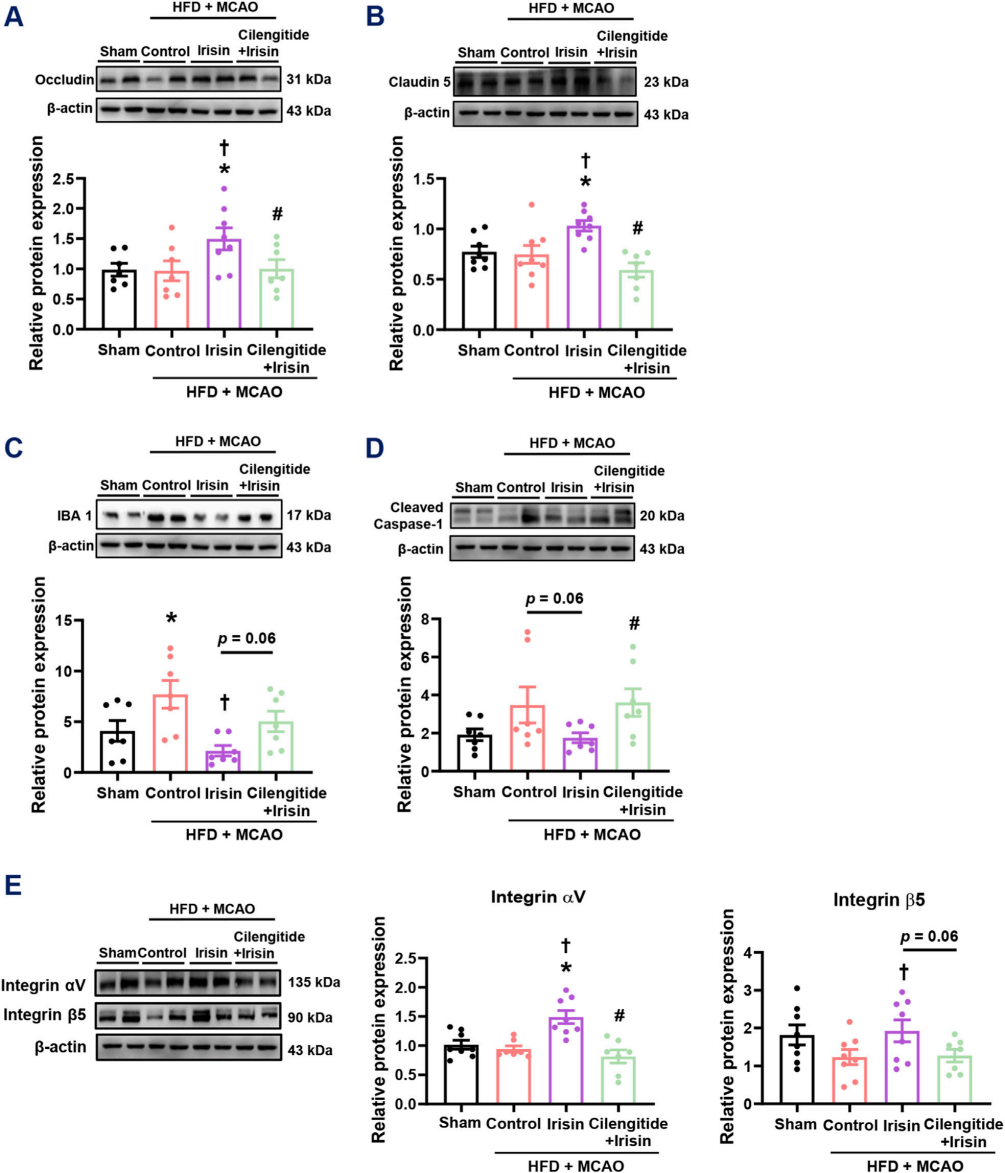
The impact of integrin αV/β5, an irisin receptor, on ischemic stroke outcomes. Cilengitide, an inhibitor of integrin αV/β5, was administered (10 mg/kg, intraperitoneally) 2 hours prior to MCAO surgery, and recombinant irisin (750 μg/kg) was injected immediately before MCAO surgery. All MCAO mice were fed HFD for 8 weeks prior to surgery. **(A–E)** Assessment of protein levels in the brain. Protein levels were evaluated through western blot. *p < 0.05 vs. Sham (NCD-fed mice subjected to sham surgery), ^†^ *p* < 0.05 vs. Control + HFD + MCAO, ^#^ *p* < 0.05 vs. Irisin + HFD + MCAO.

To determine whether the neuroprotective effects of irisin are mediated through a specific ligand–receptor pathway, we next examined the requirement for its known receptor, integrin αVβ5, using cilengitide as a pharmacological inhibitor (Wang et al., 2023). Consistent with our previous findings, irisin treatment markedly increased the protein expression of occludin and claudin-5. However, this effect was abolished when cilengitide was administered prior to irisin treatment (Figures 7A,B). In parallel, IBA1 protein expression was significantly reduced by irisin treatment, and this reduction was reversed by cilengitide pretreatment (Figure 7C). Similarly, the irisin-induced suppression of cleaved caspase-1 protein expression was significantly attenuated by integrin αVβ5 inhibition (Figure 7D). Interestingly, irisin administration increased the protein expression of integrin αV and β5, whereas pretreatment with cilengitide reduced integrin αVβ5 expression despite subsequent irisin administration (Figure 7E), suggesting a positive regulatory loop in which irisin-mediated receptor engagement sustains integrin αVβ5 expression. Taken together, these findings demonstrate that irisin exerts its therapeutic effects in obesity-associated ischemic stroke through integrin αVβ5–mediated signaling.

## 4 Discussion

The present study suggests exercise-induced irisin as a neuroprotective agent in an obesity-associated model of ischemic stroke. Using a multifaceted experimental approach, we demonstrate that: (1) diet-induced obesity exacerbates ischemic brain injury and reduces circulating irisin levels, whereas treadmill exercise mitigates these effects through enhanced systemic and cerebral FNDC5/irisin production; (2) recombinant irisin administration recapitulates the neuroprotective effects of exercise, reducing infarct size, preserving BBB integrity via occludin and claudin-5 upregulation, suppressing microglial activation, promoting M2 macrophage polarization, and inhibiting NLRP3 inflammasome-driven pyroptosis at the protein level; (3) FNDC5 deficiency exacerbates the pathological phenotype in obese MCAO mice, with a concomitant reduction in integrin αVβ5 receptor expression; and (4) pharmacological blockade of the integrin αVβ5 receptor with cilengitide abolishes the neuroprotective effects of irisin, establishing the receptor-dependent nature of irisin’s actions in ischemic stroke. Collectively, these findings support a model in which irisin protects the metabolically compromised ischemic brain through integrin αVβ5-dependent preservation of neurovascular integrity and suppression of neuroinflammation.

Obesity is a major modifier of ischemic stroke outcome, in part through chronic systemic inflammation, endothelial dysfunction, and impaired neurovascular homeostasis (Kernan et al., 2013). As the global obese population continues to expand rapidly (Collaboration, 2024), obesity-driven metabolic dysfunction is increasingly recognized as a primary and growing contributor to both stroke incidence and exacerbated clinical outcomes (G.B.D.S. Collaborators, 2021; Chen et al., 2018; Bushnell et al., 2024). Investigating stroke in the context of obesity is crucial, as conventional preclinical models utilizing healthy, lean animals often fail to recapitulate the complex pathophysiology of stroke patients, who frequently harbor metabolic comorbidities (Le Thuc and Garcia-Caceres, 2024; Ramirez-Carreto et al., 2023). By utilizing an HFD-induced obese stroke model, our study directly addressed this critical translational gap. Consistent with the detrimental effects of metabolic stress, HFD-fed mice in our study exhibited larger infarct volumes and poorer early survival after MCAO than lean controls. However, treadmill exercise effectively attenuated these adverse outcomes and restored MCAO-reduced circulating irisin levels, demonstrating the profound impact of metabolic intervention on stroke pathology.

Importantly, our data revealed that exercise increased FNDC5/irisin expression in skeletal muscle alongside a parallel upregulation in the brain, suggesting that both peripheral and central FNDC5/irisin responses may contribute to exercise-mediated neuroprotection. These results are particularly relevant because most previous studies of irisin in cerebral ischemia were performed in lean or metabolically normal animals (Asadi et al., 2018; Li et al., 2017; Guo et al., 2019). The present study extends this literature by showing that irisin remains protective in an obesity-associated ischemic environment, where endogenous irisin signaling appears to be impaired and the brain is already pre-conditioned toward an exaggerated inflammatory state (Le Thuc and Garcia-Caceres, 2024; Ramirez-Carreto et al., 2023). Supporting the functional importance of endogenous FNDC5/irisin, exogenous irisin administration to FNDC5 KO mice attenuated microglial activation and inflammasome activation, indicating that the aggravated ischemic phenotype of FNDC5-deficient mice is, at least in part, attributable to the loss of irisin itself. Together, these loss- and gain-of-function data position endogenous FNDC5/irisin as a tractable target in the metabolically compromised brain.

Prior work has progressively delineated the neuroprotective repertoire of irisin in cerebral ischemia. In lean MCAO rodents, systemic administration of recombinant irisin reduces infarct volume, improves neurological deficits, and activates Akt and ERK1/2 pro-survival signaling, establishing irisin as a *bona fide* neuroprotective myokine (Li et al., 2017). Subsequent studies showed that irisin attenuates ischemia/reperfusion-induced neuronal apoptosis through preservation of mitochondrial function (Asadi et al., 2018) and protects BBB integrity by inhibiting matrix metalloproteinase-9 (MMP-9)-mediated degradation of neurovascular basement membrane components (Guo et al., 2019). Although these studies firmly established irisin’s neuroprotective profile, causal linkage to exercise-induced protection has so far rested on a neutralizing antibody approach in lean animals. Our study advances this framework by combining genetic FNDC5 deficiency, recombinant irisin administration, and receptor-level pharmacological blockade, providing a multi-layered, convergent validation of the FNDC5/irisin/integrin αVβ5 axis. Within this context, we extend BBB protection to the obesity-ischemia setting by showing that irisin upregulates occludin and claudin-5 in HFD-fed MCAO mice. Because tight junction disruption drives vasogenic edema, immune infiltration, and secondary neuronal injury, this convergence across models and metabolic states establishes irisin-mediated BBB protection as a robust and reproducible mechanism. Whereas prior work emphasized MMP-9–mediated matrix degradation, our findings implicate direct maintenance of endothelial tight junction proteins as a complementary, parallel BBB-protective pathway.

The neuroinflammatory response following ischemic stroke is shaped by microglial activation and macrophage polarization, with a sustained M1-dominant phenotype driving tissue injury and an M2-dominant phenotype supporting resolution and repair. In the present study, irisin significantly reduced IBA1 expression in the ischemic brain of HFD-fed MCAO mice, indicating attenuation of microglial activation. Concurrently, irisin upregulated canonical M2 macrophage markers, including CD206 and YM1/2, without significantly altering M1 markers in HFD + MCAO mice, consistent with a selective shift toward an anti-inflammatory, reparative phenotype rather than global immune suppression. A reduction in M1 markers was, however, observed in irisin-treated sham mice under non-ischemic conditions, suggesting that irisin may attenuate baseline pro-inflammatory macrophage tone independently of ischemic injury, though its functional relevance requires further investigation. This pro-M2 polarization aligns with reports in intracerebral hemorrhage, where irisin inhibited M1 microglia/macrophage polarization and promoted the M2 phenotype via integrin αVβ5/AMPK signaling (Wang et al., 2022a), and in a postoperative cognitive dysfunction model, in which irisin promoted microglial M2 reprogramming through the integrin αVβ5/STAT6 pathway (Wang et al., 2025).

Beyond macrophage polarization, irisin suppressed NLRP3 inflammasome–driven pyroptosis, a key effector of secondary injury after ischemic stroke (Fusco et al., 2020). Pyroptosis is a gasdermin-driven, lytic and highly pro-inflammatory form of programmed cell death, where inflammasome assembly activates caspase-1, cleaves gasdermin D (GSDMD) into its pore-forming fragment and matures IL-1β and IL-18, thereby amplifying neurovascular inflammation and tissue loss in the ischemic brain (Wang L. et al., 2022). Canonical NLRP3 activation proceeds in two discrete steps—a transcriptional “priming” signal that induces NLRP3 and pro-IL-1β, followed by a post-translational “activation” signal that drives caspase-1-mediated GSDMD cleavage (Swanson et al., 2019). Obesity itself constitutes a chronic priming stimulus–lipotoxic danger signals such as ceramide sustain NLRP3 activation (Vand et al., 2011) – and in obese rodents this pre-primed state predisposes the ischemic brain to exaggerated NLRP3/GSDMD-mediated pyroptosis (Zhao et al., 2024). Against this background, our observations–a reduction in cleaved caspase-1 and GSDMD protein without altering NLRP3 or other inflammasome-component mRNA–are consistent with this two-step framework in a clinically relevant, metabolically compromised *in vivo* setting, suggesting that irisin acts predominantly at the post-translational activation step rather than by repressing transcriptional priming. These results align with prior reports that irisin restrains ROS-dependent NLRP3 activation in oxygen-glucose deprivation (Peng et al., 2017) and lean ischemic stroke (Liu et al., 2020), as well as in traumatic brain injury (Zhang et al., 2025), Parkinson’s disease (Cai et al., 2026), and exercise-driven hippocampal inflammasome suppression (Zhao et al., 2025) – while, to our knowledge, identifying reduced cleaved GSDMD as a neuroprotective endpoint of irisin in obesity-associated ischemic stroke for the first time.

The causal role of integrin αVβ5 in irisin-mediated neuroprotection was directly confirmed using cilengitide, a selective cyclic RGD peptide inhibitor of integrin αVβ5 (Wang et al., 2023). Cilengitide abolished each of the salient neuroprotective endpoints of irisin in our model—BBB preservation, M2 polarization, and suppression of cleaved caspase-1 — establishing integrin αVβ5 engagement as indispensable for irisin action in the obese ischemic brain. In other injury contexts, cilengitide similarly abolished irisin’s anti-neuroinflammatory and anti-apoptotic effects in intracerebral hemorrhage (Wang et al., 2022a); integrin β5 knockdown eliminated irisin’s cardioprotective and anti-inflammatory activities in hemorrhage-induced organ injury (Wang et al., 2023); and in acute glaucoma, integrin αVβ5/AMPK signaling served as the mechanistic hub for irisin-driven microglial M2 conversion and autophagy induction (Zhang et al., 2024). In line with these reports, we propose integrin αVβ5 as the principal signaling nexus through which irisin orchestrates multi-target neuroprotection in the ischemic brain, though research on its downstream effectors warrants direct interrogation in future work.

A set of interrelated observations points to a potential feed-forward regulatory loop between irisin and its receptor. First, exogenous irisin increased FNDC5 mRNA and showed a trend toward elevated FNDC5 protein in the brains of HFD + MCAO mice, indicating that irisin may upregulate its own precursor. Second, integrin αV and β5 protein levels were markedly reduced in FNDC5 KO brain, indicating that endogenous FNDC5/irisin signaling is required to maintain basal receptor expression. Third, irisin-induced integrin αV and β5 upregulation was prevented by cilengitide, indicating that receptor induction is itself integrin αVβ5-dependent. Together, these findings are compatible with a feed-forward arrangement in which irisin–integrin αVβ5 engagement reinforces both ligand and receptor availability, potentially sustaining neuroprotective signaling beyond the initial exposure. A plausible mechanistic basis is that irisin–integrin αVβ5 activates AMPK/PGC-1α to drive neuronal FNDC5 expression via the canonical PGC-1α/FNDC5 axis (Bostrom et al., 2012; Wrann et al., 2013). More broadly, the reduced basal integrin αVβ5 in FNDC5 KO brain supports the view that endogenous irisin constitutes a tonically active neuroprotective signal whose loss under physical inactivity or obesity may progressively sensitize the neurovascular unit to ischemic insult by attenuating both ligand production and receptor availability.

In conclusion, the present study establishes that exercise-induced irisin is a critical neuroprotective mediator in obesity-associated ischemic stroke, acting through integrin αVβ5-dependent signaling to preserve BBB integrity, promote anti-inflammatory macrophage polarization, and suppress NLRP3 inflammasome-mediated pyroptosis at the protein-activation level. The convergent genetic, pharmacological, and receptor-level evidence presented here positions the FNDC5/irisin/integrin αVβ5 axis as a therapeutically actionable target under metabolically compromised conditions—a context in which current reperfusion therapies offer limited benefit and circulating irisin is chronically suppressed (Wu et al., 2019). These results hold significant implications for the field of stroke research, underscoring the necessity of moving beyond generalized neuroprotective paradigms toward comorbidity-specific therapeutic strategies. As the global prevalence of obesity continues to rise, priming the brain for exaggerated injury and limiting the efficacy of endogenous recovery mechanisms, identifying targets such as the FNDC5/irisin axis that specifically counteract metabolic neuroinflammation is increasingly crucial (Le Thuc and Garcia-Caceres, 2024). From a translational standpoint, the demonstration that a single intraperitoneal dose of recombinant irisin confers significant neuroprotection in a clinically relevant obesity-stroke model provides proof-of-concept supporting its development as a neuroprotective agent. Future investigations should focus on elucidating the downstream signal transduction cascade of integrin αVβ5, optimizing the post-stroke therapeutic window, and evaluating the long-term neurorestorative potential of irisin in preclinical and clinical settings.

## Data Availability

The original contributions presented in the study are included in the article/supplementary material, further inquiries can be directed to the corresponding authors.
